# Propagation Modeling and Defending of a Mobile Sensor Worm in Wireless Sensor and Actuator Networks

**DOI:** 10.3390/s17010139

**Published:** 2017-01-13

**Authors:** Tian Wang, Qun Wu, Sheng Wen, Yiqiao Cai, Hui Tian, Yonghong Chen, Baowei Wang

**Affiliations:** 1 School of Computer Science and Technology, Huaqiao University, Xiamen 361021, China; 1400214020@hqu.edu.cn (Q.W.); caiyq@hqu.edu.cn (Y.C.); htian@hqu.edu.cn (H.T.); iamcyh@hqu.edu.cn (Y.C.); 2 College of Information Technology, Deakin University, Melbourne, VIC 3125, Australia; sheng.wen@deakin.edu.au; 3 School of Computer and Software, Nanjing University of Information Science & Technology, Nanjing 210044, China; wang@nuist.edu.cn

**Keywords:** WSANs (wireless sensor and actuator networks), mobile sensor worm, modeling, patch, mobile patcher

## Abstract

WSANs (Wireless Sensor and Actuator Networks) are derived from traditional wireless sensor networks by introducing mobile actuator elements. Previous studies indicated that mobile actuators can improve network performance in terms of data collection, energy supplementation, etc. However, according to our experimental simulations, the actuator’s mobility also causes the sensor worm to spread faster if an attacker launches worm attacks on an actuator and compromises it successfully. Traditional worm propagation models and defense strategies did not consider the diffusion with a mobile worm carrier. To address this new problem, we first propose a microscopic mathematical model to describe the propagation dynamics of the sensor worm. Then, a two-step local defending strategy (LDS) with a mobile patcher (a mobile element which can distribute patches) is designed to recover the network. In LDS, all recovering operations are only taken in a restricted region to minimize the cost. Extensive experimental results demonstrate that our model estimations are rather accurate and consistent with the actual spreading scenario of the mobile sensor worm. Moreover, on average, the LDS outperforms other algorithms by approximately 50% in terms of the cost.

## 1. Introduction

Wireless sensor and actuator networks (WSANs) have a broad range of applications, such as environmental monitoring [1], military surveillance [2,3], object tracking [4,5], etc. In typical WSAN architecture, there are several actuator elements with a certain mobile capability. For a very practical example, in a smart agriculture system, there are usually some mobile tractors monitoring and gathering sensing information, including temperature, humidity, etc., from distributed sensor networks [6]. Unlike ordinary sensors that are stationary after initial deployment, these mobile units are capable of moving around (passively or actively) and performing interactive tasks efficiently. However, the mobility of these particular elements also incurs new security risks, which are usually neglected by researchers (e.g., mobile worm attacks). 

Worm attacks are always the most imminent and effective threats against energy, information confidentiality, and service availability in various applications of wireless sensor networks (WSNs) [7,8]. In general, sensors are employed in unattended environments and equipped with simple hardware architecture, low memory, and computational resources. These limitations caused by the wireless nature and decentralized architecture of signal communication make security provisioning difficult; as a result, the ability of sensors to defend against the worm attack cannot meet our expectations. Even without these constraints, designing foolproof security protocols and codes is almost impossible in real life. These leave the door open to sensor worms, which take advantage of the vulnerabilities to propagate via exploiting the multi-hop message transmission mechanism. Moreover, since all sensors execute the same program image, exploiting one’s vulnerabilities can compromise all by diffusing self-replicating worm copies [9]. Therefore, sensor networks are more vulnerable to worm threats than traditional networks. Previous studies have indicated that the sensor worm attacks have become one of the major threats to WSN applications [10]. 

This is even worse in WSANs, where the actuator elements have the potential to be mobile worm carriers and assist in diffusing worm copies. Through simulations, we demonstrate that once an attacker successfully compromises an actuator and makes it a mobile worm carrier, the overall worm dissemination process is considerably accelerated. Actually, it is not only the change in the worm spreading velocity, but also the increase in complexity of worm transmission behavior (the shape of the infection region caused by the worm infection is extremely irregular and cannot be described by differential equations.). In [11], Ho et al. noted that as a more destructive attack pattern, the newly-emerging mobile worm attacks are becoming attractive, and even essential, for the attackers.

Although a few studies have considered mobile worm attacks, existing modeling and defending techniques are not practical for this exceptive worm attack pattern. First, for modeling the propagation dynamic, previous models are almost all derived from epidemic models that are completely based on differential equations. These series of propagation models can only provide macroscopic estimation of the static sensor worm’s infection scale and cannot accurately estimate the infection scale of the mobile one. Second, the mobility of the worm carrier makes the worm’s spreading behavior more complicated. Traditional worm defense methods do not consider the mobile worm carrier, and they are not applicable to defending against it. On the one hand, mobile actuator elements make the network topology change constantly, which does not satisfy the implementation conditions of the traditional immunization algorithms that aim at the static network topology. On the other hand, previous algorithms do not have a directional immunization operation on the mobile worm carrier. This leads to frequent emergence of new infection areas that cannot be immunized on time. Therefore, it is necessary to design a new defending method that considers the microscopic propagation behavior of a mobile worm carrier.

In this paper, we propose modeling the propagation dynamic of a mobile sensor worm by several iterative equations of individual security states from the microscopic point of view. The model follows the state transition scheme of a typical susceptible-infected (S-I) infection model, but can microscopically compute the prior probability of each sensor being infected by the worm. Unlike the prior methods, we design a mixed defense strategy, including both blocking and recovering operations. As a two-step method, we first obtain the estimated infection boundary by employing the convex hull theory and then temporarily make peripheral sensors of the convex hull sleep to block the worm’s further spreading. Second, we implement a mobile patcher to recover infected sensors by distributing patches in a determined convex region and, thus, minimize the cost. Our major contributions are listed below:

- We propose a microscopic propagation model for a mobile sensor worm to describe its propagation dynamic. This model can estimate the individual state, which is distinguished from traditional global models.
- We carry out a series of experiments to evaluate the validity of the proposed propagation model. The experiments are based on WSANs with different scales. The results show that the proposed analytical model is rather accurate compared with the real infection scenario.
- We design a two-step local defending strategy (LDS) to defend against the mobile sensor worm efficiently. Based on the estimation of the infection boundary, we implement a mobile patcher to recover infected sensors at a low cost. Robustness and efficiency of our methods are validated through extensive analyses and experiments.

The rest of the paper is organized as follows: Section 2 introduces related work and summarizes the shortcomings of the existing research. Preliminary research and some assumptions are introduced in Section 3. In Section 4, we model the propagation dynamic of a mobile sensor worm. An efficient defending strategy is proposed to deal with the mobile sensor worm from the microscopic point of view in Section 5. Performance analyses are conducted in Section 6. Extensive experimental results are demonstrated in Section 7. At the end, Section 8 concludes the full paper.

## 2. Related Work 

### 2.1. Worm Attack in Networks 

Computer worms have been a persistent security threat on the Internet since the first Morris worm arose in 1988 [12]. It is usually a self-replicating program (or a set of program) that can spread its own copies or some parts of itself into other computer systems through the network. In general, worms can attack computers independently without users’ intervention, with a huge destructive power to cyber security. Computer worms that have appeared, such as Red Code and Slammer, once attacked a large number of hosts successfully with specific vulnerabilities in a very short period of time, resulting in large economic losses [13]. Patch-management solutions are usually feasible to defend against the worm storms and recover the infected hosts in traditional TCP/IP networks [14]. However, it requires additional software, testing infrastructure, and sitewide policies to deploy. Even if we make it, the deployment cost is very large. Some other studies proposed that the anti-worm (i.e., white worm) can be an effective means of counterattack. It is utilized to spread disinfection codes to the sensors’ firmware as the same transmission mode as a black worm [15]. Unfortunately, Nicholas et al. observed that anti-worms cannot work well on the Internet while considering legality and technical feasibility [16]. As a result of this, the development of anti-worm technology is restricted in traditional TCP/IP networks. 

### 2.2. Sensor Worm in WSNs

Recent advances have shown that attackers are capable of launching worm attacks that target compromised sensors with or without physical contacts in WSNs. A concrete method is to exploit certain types of vulnerabilities of sensors, such as buffer overflows. Since all sensors execute the same program image, exploiting such vulnerabilities can compromise all sensors by diffusing self-propagating worm packets [10,17]. Sensor worm attacks over static WSNs are extremely destructive due to a large amount of generated scanning and communication traffic. This may cause serious problems, such as channel blocking and energy exhaustion. Sensor worm attacks have become one of the major threats to the applications of WSNs [18,19].

In response to threats caused by the sensor worm, researchers have done some worthwhile work. For example, Hosseini et al. utilized a software diversity approach to defend against sensor worm attacks by minimizing the total number of defective edges with limited software versions [20]. Shen et al. formulated a sensor worm defense differential game to dynamically choose strategies and, thus, minimize the overall cost [21]. The core principle of these methods is to select a certain proportion of sensor nodes and perform immunization operations, like patching, on them. Most of these methods consider the degree characteristic as the measure standard. However, these methods do not consider the mobile worm carriers, and they are not applicable to defending against it. 

Several recent papers [22,23] considered that anti-worm technology is feasible in WSN because of its particularity different from the traditional TCP/IP networks. First, sensors are usually owned by the same entity, which means that we need not consider most legal problems. Second, as we know, sensors are usually equipped with particularly simple software and hardware architecture, low memory, and computational resources, which make it easy for them to be compromised by malicious worms, as well as the anti-worm. However, a simple anti-worm does not solve the excessive traffic problem as the anti-worm code usually has to spread over the whole network and causes massive traffic consumption [24]. Furthermore, existing anti-worm technology may not be mature enough to quickly generate anti-worm code after specific black worms are detected. Therefore, to be practical, we propose implementing a mobile patcher rather than an anti-worm to recover the infected sensors in this paper. Patching solutions are also employed to defend against the sensor worm by some other papers [21,22]. In practical applications, once the mobile sensor worm starts spreading, after a certain amount of time, it is detected by the network owner, and then a patch code is developed for it. This patch is injected into the network like a new firmware or software update and spreads similar to a black worm in a restricted region. 

Similar to worm attacks on the Internet, those in WSNs start slow, but eventually achieve exponential propagation during the attack process [25]. In an effort to combat the sensor worm more effectively, it is critical to understand its propagation behavior accurately. Therefore, scientists proposed and evaluated a series of mathematical and simulation models. Among them, Tang proposed an improved SI infection model by introducing a sleeping mechanism for sensors on the basis of classical epidemic theory [26]. Mishra et al. delved into the pathogenesis of the sensor worm and proposed an SIRC (susceptible-infectious-recovered-crashed) model considering sensor nodes’ breakdown caused by infection [27]. Focusing on the temporal and spatial dynamics of sensor worm diffusion, Feng et al. utilized a differential dynamic theory, according to the energy consumption and distribution density of sensor nodes, to describe the propagation dynamics, qualitative analysis and stability of communication of the sensor worm [9]. 

However, there are some serious shortcomings in these global models. On the one hand, these models have not considered the existence of the mobile worm carriers in WSANs. The implementation of worm attacks on the mobile elements is feasible and worthwhile to the adversary, and mobile carriers have the potential to accelerate the whole diffusion process. On the other hand, most of these studies utilized epidemic models by making some assumptions which are not very realistic in WSNs. For example, Tang et al. assumed that every sensor entity has an equal chance, per unit of time, of coming into contact with every other [26], which is not practical in sensor networks. Zou et al. demonstrated that these models have relatively low accuracy through a high number of experimental simulations [28]. Unlike these global models, it is worth mentioning that Haghighi et al. proposed a bottom-up individual spreading model to describe dynamic of worm in statc WSNs [22]. However, this model only aimed at the circular or rectangular network boundary, so it has a poor scalability. Moreover, this model is also unable to describe the worm diffusion scenario with mobile carriers in WSANs.

Currently, there are a few studies about mobile worm attacks in WSANs. Among them, Ho et al. introduced the problem of mobile malicious nodes in WSNs and proposed a distributed detection method [11]. The key principle of the proposed scheme is to apply sequential hypothesis checking to discover sensors that are silent for an unusually large number of time periods. The authors also studied worm propagation patterns of mobile worm carriers in mobile sensor networks and proposed mobile-to-mobile models [29,30]. Valler et al. noted that mobile devices are capable of being tipping points for worm breakout [31]. These studies all call attention to the feasibility and fatalness of mobile worm attacks [32]. However, they did not provide appropriate spreading models to represent the propagation behavior of the mobile sensor worm or effective methods to defend the mobile sensor worm.

## 3. Preliminary Assumptions

We assume that numerous sensors are densely deployed in a two-dimensional surveillance region with the deployment density δ nodes/m^2^. A mobile actuator moves randomly, that is, it moves in one direction before turning in another direction after some random time τ called the direction delay, with a velocity *v*. According to the experiments in [33], worm infection always have a time delay α, which includes the time receiving and forwarding the worm packets, as well as the restart time of the infected sensor caused by the infection. The infection rate, denoted by β, is the probability that each infectious node passes the worm copies to a susceptible neighbor over a unit of time. The infected sensor nodes deliver worm copies to their neighbors by repeating the same process.

**Figure 1:**
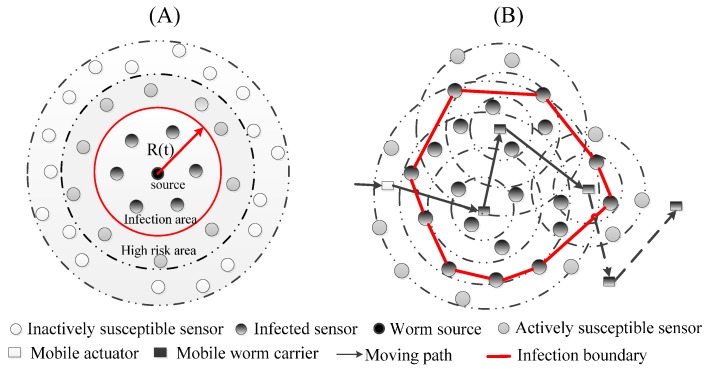
Static sensor worm (**A**) vs. Mobile sensor worm (**B**).

In Figure 1, the circles and rectangle represent fixed sensors and the mobile actuator, respectively. Suppose that the mobile worm carrier starts to spread worm copies at time *T =* 0. In this exemplification, the black nodes are the *infected sensors*. Grey nodes represent the healthy neighbors of infected nodes, which are referred to as *actively susceptible sensors*. White nodes are healthy sensors without unhealthy neighbors, which are called *inactively susceptible sensors*. After some spreading time *T*, the infection region, referred to as the *infection boundary*, is the area bounded by the red lines. We assume that an attacker can launch a mobile worm attack by physically capturing the mobile actuator, or just remotely compromising it by exploiting software vulnerabilities [9]. The mobile sensor worm can be detected by the methods introduced in [11], and then a targeted patch is developed for it on time. Then this patch is injected into the network to recover the infected sensors. We assume that the energy consumed by one sensor during a unit time is equal to *e* and will increase *ϕ* percent once the sensor is infected by the worm. Accordingly, when there are *I(t)* infected sensors in the network, the energy consumption of networks with infected sensor nodes per unit time can be represented by:

(1) $$\begin{array}{ll} E & =\left(N-I\left(t\right)\right)*e+I\left(t\right)*e*\left(1+\varphi \right)\\ & =\left(N+I\left(t\right)\varphi \right)*e \end{array}$$

We note that an authentication mechanism is very common in WSNs to prevent unauthorized and corrupted messages being forwarded. Since this is not the core work of this paper, we simply employ Subha’s authentication mechanism MES [34] as the supplement to the original method. MES consists of three algorithms: a key generation algorithm, a signature algorithm, and a verification algorithm. With the aid of this authentication strategy, we can significantly reduce the attack ability of worms [35]. In addition, we also simply consider mobile elements higher-value targets that must be guarded more closely. In general, mobile devices have stronger hardware and software equipment and energy than ordinary sensors. They can install more defense software so we consider them less vulnerable, compared with normal resource-constrained sensors. However, the mobile elements may be prime targets from an attacker’s view. If a mobile element is captured by a worm virus, the actuator’s mobility can causes the worm to spread faster.

With the aid of the authentication mechanism and these hypotheses of the mobile element, the proposed LDS solution with a mobile patcher can work in WSANs. Note that we focus on how to spread the patches to those infected sensors and how to design it is out of the study scope of this paper. In other words, we try to provide the infected sensors with “medicine” and expect it can cure the infected sensors. With the improvement of software technology and the decrease of hardware cost, creating a patch against a discovered loophole quickly can be achieved. For example, a patch/anti-worm can be designed quickly with cloud-computing technologies which are out of the WSN and then is injected into the WSAN [36]. It is worth noting that even if the worm blocking fails, the mobile patcher can still be employed to “treat” the infected sensors.

## 4. Propagation Dynamic of the Mobile Sensor Worm

**Figure 2:**
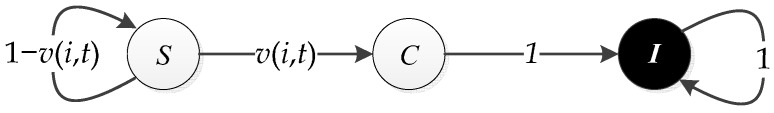
The state transition graph of a sensor node in worm propagation.

Zou et al. observed that the traditional worm diffusion models based on epidemic theory largely overestimate the infection scale of Internet worms, and it is also true in WSANs [28]. Although there are other individual models, such as [22], they did not solve the challenges caused by mobile worm carriers. To this end, we propose a new mathematical model, from the microscopic view, to model the diffusion dynamics of a mobile worm carrier. This model follows the state transition scheme of typical susceptible-infected (S-I) infection models but can microscopically compute the prior probability of each node being infected. As shown in Figure 2, there are three states in this model: “S” indicates the susceptible state, “C” indicates the contagious state, and “I” indicates the infected state. The “S” state transits to the “C” state with probability v(i,t). We use $P_{S}\left(i,t\right)$, $P_{C}\left(i,\;t\right)$, and $P_{I}\left(i,\;t\right)$ to denote the probability of node *i* being susceptible, contagious, and infected at time *t*, respectively. Moreover, v(i,t) denotes the probability of node *i* being converted from “S” to “I”. We then have the following iterations:

(2) $$P_{S}\left(i,\;t\right)=[1-v\left(i,t\right)]P_{S}\left(i,t-1\right)$$

(3) $$P_{C}\left(i,t\right)=v\left(i,t\right)P_{S}\left(i,t-1\right)$$

(4) $$P_{I}\left(i,t\right)=v\left(i,t\right)P_{S}\left(i,t-1\right)+P_{I}\left(i,t-1\right)$$

**Figure 3:**
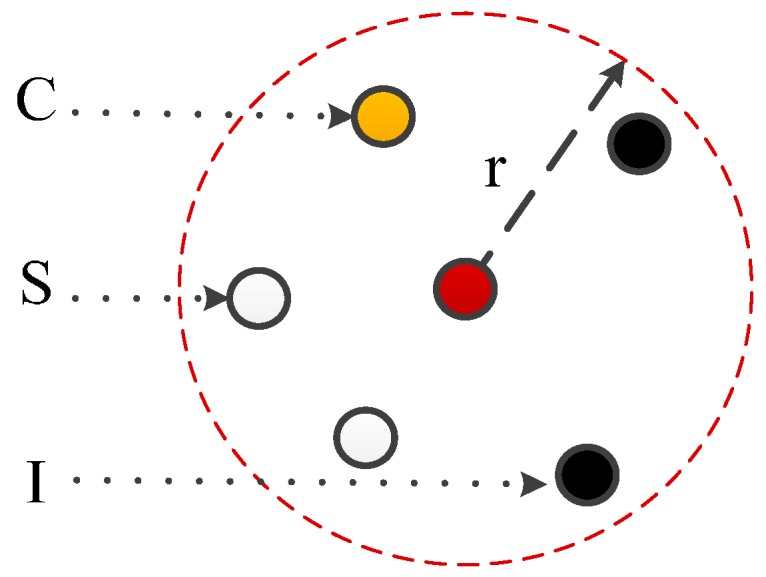
Microscopic view of a sensor individual in propagation.

In Equation (4), the value of v(i,t) is the probability of the sensor *i* being infected by its single-hop neighbors at time *t*. Normally, a sensor can infect its neighbors only when it is in a contagious status [37]. The variable $\gamma_{ij}$ is the propagation probability between sensor *i* and sensor *j* ($\gamma_{ij}\in$ [0,1]). If $\gamma_{ij}=0$, sensor *i* has no connection with sensor *j*. As shown in Figure 3, the security status of sensor individual at time *t* is relevant to its contagious neighbors (yellow ones). Therefore, according to the principle of multiplication, we have:

(5) $$v\left(i,t\right)=1-\underset{j\in N_{i}}{\prod }\left[1-\gamma_{ji}P_{C}\left(j,t-1\right)\right]$$

## 5. Local Area Defending Algorithm

In this section, to combat against the mobile sensor worm, we propose a defense strategy that can be divided into two steps. The first step is to estimate the worm infection region and prevent its further spreading. In the second step, we implement a mobile patcher to recover the infected sensors. Note that the infected mobile actuator should be repaired first to prevent the emergence of new infected regions. We design an algorithm for bounding the infection region in Section 5.1, and a detailed defending strategy is presented in Section 5.2. In Section 6, some performance analyses are carried out.

### 5.1. Bounding the Infected Area of the Mobile Worm

We consider investigating the mobile sensor worm’s infection dynamics in the locations where the mobile actuator changes its moving direction (called turning direction point, or TDP for short). The outermost sensors of every TDP’s infection region are picked out and put into a candidate set. Next, we employ the convex hull theory to acquire all outermost sensors in the global field of vision from the candidate set.

We first bound the worm infection area of a static sensor worm. Figure 1A presents the propagation scenario of a static sensor worm. Starting from the worm source, the infection area expands a radius length every time tick. Thus, when the propagation time is equal to *T*, the maximum infection radius of the static sensor worm can be obtained approximately by the following formula:

(6) $$r\left(T\right)\approx \frac{T}{\alpha }*r$$

In light of the propagation dynamic of the static sensor worm, we can calculate the maximum infection radius of ith TDP by the following formula:

(7) $$Ir_{i}\left(T\right)\approx \frac{T-i\tau }{\alpha }*r$$

We then calculate distances between every sensor to all TDPs. If the distance is greater than $Ir_{i}\left(T\right)-r$ and less than $Ir_{i}\left(T\right)-r$, we judge this sensor as a marginal node and put it into a candidate set. After repeating this procedure, we can acquire all sensors that have the possibility of appearing on the infection border. Based on this, the original problem can be defined as a new problem. That is, when the locations of a group of nodes are known, how does one find their geometric boundary? 

The convex hull of a point set Q is a minimal convex polygon P, and it meets the limiting condition that all points in Q are in the interior or on the border of P. Therefore, we can obtain a fairly accurate geometric boundary of the infection area by calculating the convex hull of the candidate set. There are quite a few methods for computing the convex hull, like Graham’s scan and the Jarvis march. In this paper, we apply Graham’s scanning strategy to seek the convex hull of the infection region. Its algorithm principle is briefly introduced below. By maintaining a stack S of convex vertices set M, each point in the candidate set Q is pressed into S once, and the vertices not in M finally pop up; they are judged by the size of pole angles. At the end of Algorithm 1, the stack S contains only the vertices of M, and these vertices appear on the boundary in a counter-clockwise order. Details of the proposed algorithm are presented in Algorithm 1. Since the time complexity of obtaining the candidate set is O (N^2^), where N is the number of sensor nodes and the time complexity of Graham’s scan is O (n log n), where n is the number of the candidate points, the integral time complexity of Algorithm 1 is O (N^2^).

**Algorithm 1: Estimating the Geometry Boundary of Infected Area**Input: localizations of $\mathrm{TDPs}\;(x_{i},\;y_{i})\;$ and of all sensor nodes $\left(a_{j},b_{j}\right)$; current time T; communication radius *r*; infection delay of the sensor worm *α*; Direction delay of the mobile actuator *τ*;Output: vertex sequence of convex hull $S\langle S_{1},{\;S}_{2},\ldots S_{n}\rangle$;For positive integer *i* := 1 to $\frac{T}{\tau }$ + 1 do  Calculate the infection radius of each TDP: ${\mathrm{Ir}}_{i}\left(T\right)=\frac{T-i\tau }{\alpha }*r$// Calculate distances between all nodes to all TDPs:  For positive integer j := 0 to N−1    $D_{ji}=\sqrt{{\left(a_{j}-x_{i}\right)}^{2}-{\left(b_{j}-y_{i}\right)}^{2}}$    If $\left(Ir_{i}-r\leq D_{ji}\leq Ir_{i}\right)$ add $\left(a_{j},b_{j}\right)$ into candidate set M    End if  End forEnd forRenumber elements of the set M// seek the convex hull.Let $M_{0}$ be the point in M with the minimum *y*-coordinate or the leftmost point$\mathrm{Let}\;\langle M_{1},{\;M}_{2}\ldots {\;M}_{m}\rangle$ be the candidate points in M, sorted by polar angle in counterclockwise order around $M_{0}$Let S be an empty stackPush $\;\left(M_{0},S\right)$; Push $\;\left(M_{1},S\right)$; $\mathrm{Push}\;\left(M_{2},S\right)$;For k = 3 to m  While the angle formed by points NEXT-To-TOP(S), TOP(S), and $M_{k}$ makes a nonleft turn  POP(S); $\;\mathrm{Push}\;\left(M_{k},S\right)$;  End whileEnd forReturn S;

### 5.2. Defending the Worm with a Mobile Patcher

After acquiring the infection boundary by Algorithm 1 in Section 5.1, we are sure to prevent the worm propagation by cutting off the marginal sensors of the infection convex hull and then recovering the infected sensors in the restricted region. Note that the infected mobile actuator should be repaired first to prevent the emergence of new infected regions. Previous immunization strategies cannot handle this special mobile worm scenario. In addition, traditional algorithms usually consider the degree characteristic of the sensors as a measure of the standard, rather than immunizing the network links. They also do not take into account the network flow effect on immunization efficiency and cost. For the decentralized large-scale WSN, traditional algorithms have the problem of low efficiency and long immunization time. In our proposed LDS, we expect to immunize the sensors on the transmission paths to the outside of the determined infection region to restrain the worm from further spreading and, thus, to minimize the cost. Since the LDS can stop the increase of the infection region immediately, it is faster and more efficient than traditional immunization strategies based on the degree characteristic.

As for the defending operation, in real life, since physically patching every single sensor is not possible, recovery is usually done by patching or removing the worm/virus with a piece of anti-malware code [22]. Recently, some studies have indicated that the anti-worm (i.e., white worm) can be an effective mean of counterattack. However, the anti-worm may incur large amounts of extra scanning and message traffic as the malicious worm does if it spreads over the entire network. Furthermore, existing anti-worm technology may not be mature enough to quickly generate anti-worm code after specific black worms are detected. In this paper, thus, we propose transmitting the patches in the local region to minimize the cost and ensure the proposed method more practical. Some other papers also employ patches to defend against the sensor worms [21,22].

In our scenario, once the mobile sensor worm starts spreading, after a certain amount of time, it is detected by the network owner, and then a patch code is developed for it. Then the patch is injected into the network like a new firmware or update and distributes similar to a black worm. As for the detection algorithm for the mobile sensor worm, readers can refer to the work of Ho et al. [11]. Note that, in this paper, we do not discuss the designing details of the patch, and we mainly focus on its deployment locations and propagation manner from the sensor-level. The issue of how to implement the patch is also studied.

In terms of the concrete defense method, we propose a two-step local defending strategy (LDS) mixing the blocking and recovering measure. The core principle of our method is to determine a high-risk region (sensors in this region are very likely to be infected in the next time unit if there are no immunization or patching operations), and temporarily make peripheral sensors of the convex hull sleep to block the worm’s further spreading; then, the patch is implemented in the estimated infection region to defend against the worm. Details of the proposed strategy are presented in the following two steps.

**STEP** **1:** Obtain the infection convex hull by Algorithm 1, then cut off all network links to the convex hull by making peripheral sensors of the convex hull sleep.
**STEP** **2:** Develop and implement the corresponding patches into the infection region to recover the infected sensor nodes.

**Figure 4:**
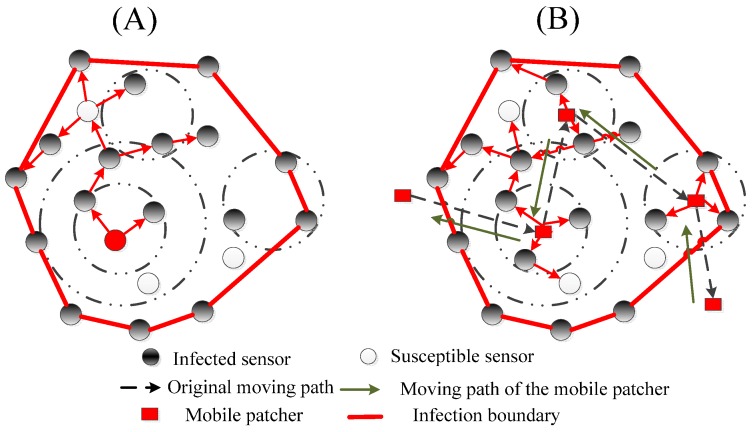
Static patch pattern (**A**) vs. mobile patcher pattern (**B**).

With regard to **STEP 2**, there may be various employment patterns, and we design two patterns for propagating the developed patch and compare their performance. In one, the patch is started from the initial infection node (in Figure 4A); while in the other one, we utilize a mobile actuator to assist in distributing patches (in Figure 4B). We refer to the first employment pattern as a static patch and the second one as a mobile patcher. The second pattern aims at using the mobile element to assist in recovering networks faster. Due to congenital defects or destruction caused by the worm, the network may not be connected very well. Under this circumstance, it is difficult for the static patch to spread over the whole infection region and recover infected sensors because the network structure may be fragmented. Mobility of the actuator can solve the recovering failure caused by the network disconnection. As shown in Figure 4B, the actuator is scheduled to return to the original moving path in the opposite direction, and it broadcasts the patches at the location of each TDP. In this mobile-assisted way, we can make sure that all infected sensors in the infection region get repaired.

## 6. Analyses

In this section, we perform some basic performance analyses for our methods. Specifically, some key indicators are calculated to demonstrate the performance of the proposed schemes. We suppose the infection convex hull obtained by Algorithm 1 has N vertices, and the locations of the vertex sequence of the convex hull are $S_{i}\;\left(x_{i},y_{i}\right)$, where i∈[1,N]; then, we obtain the following statement theories:

**Theory** **1.** The number of nodes contained within the convex hull is $\frac{N}{2*\;S\_Area}\left(\left|\begin{array}{ccc} x_{1} & y_{1} & 1\\ x_{2} & y_{2} & 1\\ x_{3} & y_{3} & 1 \end{array}\right|+\left|\begin{array}{ccc} x_{1} & y_{1} & 1\\ x_{3} & y_{3} & 1\\ x_{4} & y_{4} & 1 \end{array}\right|+\ldots \left|\begin{array}{ccc} x_{1} & y_{1} & 1\\ x_{N-1} & y_{N-1} & 1\\ x_{N} & y_{N} & 1 \end{array}\right|\right)$, where N is the number of sensor nodes in the networks and S_Area is the area of the total monitoring region.

**Figure 5:**
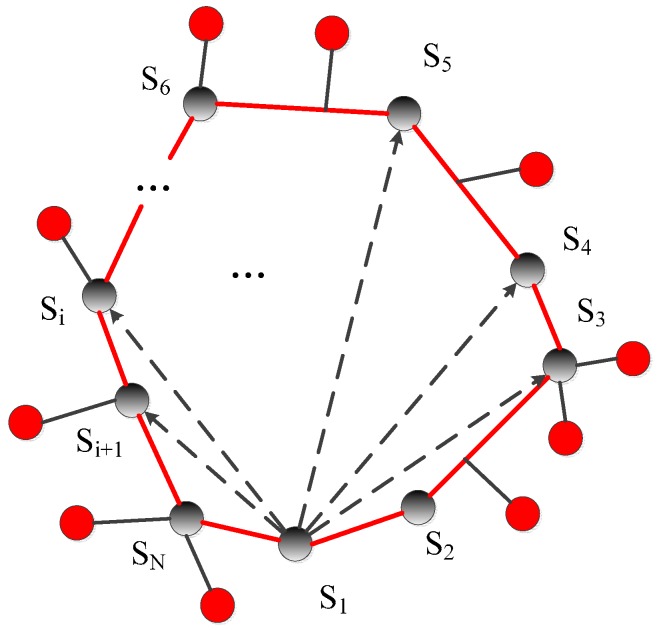
The determined convex hull obtained by Algorithm 1.

**Proof.**  Define the number of nodes within the convex hull as N_CONVEX. Since the sensor nodes are randomly deployed, we consider that the sensors obey a uniform distribution. Then we have:

(8) $$N_{\mathrm{CONVEX}}=S*\frac{N}{S\_\mathrm{Area}}$$

where S is the area of the convex hull. Since N and S_Area are the known quantities, all we have to do is to calculate the area of the convex hull. In Figure 5, we divide the convex hull into N− 2 triangles to calculate the convex area. □

We utilize $\Delta S_{i}S_{j}S_{k}$ to represent the area of the triangle $S_{i}S_{j}S_{k}$, and then, according to the knowledge of analytic geometry, we have:

(9) $$\Delta S_{i}S_{j}S_{k}=\frac{1}{2}*\left|\begin{array}{ccc} x_{i} & y_{i} & 1\\ x_{j} & y_{j} & 1\\ x_{k} & y_{k} & 1 \end{array}\right|$$

Then we have:

(10) $$\begin{array}{ll} S & =\Delta S_{1}S_{2}S_{3}+\Delta S_{1}S_{3}S_{4}+\ldots \Delta S_{1}S_{i+1}S_{i+2}+\Delta S_{1}S_{i+1}S_{N}\\ & =\frac{1}{2}\left(\left|\begin{array}{ccc} x_{1} & y_{1} & 1\\ x_{2} & y_{2} & 1\\ x_{3} & y_{3} & 1 \end{array}\right|+\left|\begin{array}{ccc} x_{1} & y_{1} & 1\\ x_{3} & y_{3} & 1\\ x_{4} & y_{4} & 1 \end{array}\right|+\ldots \left|\begin{array}{ccc} x_{1} & y_{1} & 1\\ x_{N-1} & y_{N-1} & 1\\ x_{N} & y_{N} & 1 \end{array}\right|\right) \end{array}$$

Putting Equation (10) into Equation (8), we then obtain Theory 1.

**Theory** **2.** *The number of immunization sensors (the red ones in* Figure 5*) is $\frac{N\left(d_{S_{1}S_{2}}+d_{S_{2}S_{3}}+\ldots d_{S_{n}S_{1}}\right)r}{S\_Area}$, where $d_{ij}$ is the Euclidean distance from sensor i to sensor j.*

**Proof.**  Define the number of immune sensors as N_IMMUNE. Since the immune sensors are sensors around the convex hull and have direct links to the convex hull, and the communication radius of the sensor is r, we have the following:

(11) $$N_{\mathrm{IMMUNE}}\approx L*r*\frac{N}{S\_Area}$$

(12) $$d_{ij}=\sqrt{{\left(x_{i}-x_{j}\right)}^{2}+{\left(y_{i}-y_{j}\right)}^{2}}$$

(13) $$\begin{array}{ll} L & =\left|S_{1}S_{2}\right|+\left|S_{2}S_{3}\right|+\ldots \left|S_{N-1}S_{n}\right|+\left|S_{n}S_{1}\right|\\ & =d_{S_{1}S_{2}}+d_{S_{2}S_{3}}+\ldots d_{S_{n}S_{1}} \end{array}$$

Putting Equations (12) and (13) into Equation (11), we obtain Theory 2. □

**Theory** **3.** The consumption by the local patches implementation is less than the consumption by the entire-network patch implementation $\left(1-\frac{S}{S\_Area}\right)*100\%$, where S is obtained by Equation (10).

**Proof.**  We define the consumption by the local patches implementation as CONS_Local, the consumption of entire-network patches implementing as CONS_Entire, consumption as *ω* when the patches transfer successfully from a node to one neighbor of the node, the average degree of sensor nodes as $\bar{d}$ and the percentage of A more than B as *η*. Then, we have:

(14) $$\eta =\frac{\mathrm{CONS}\_\mathrm{Entire}-\mathrm{CONS}\_\mathrm{Local}}{\mathrm{CONS}\_\mathrm{Entire}}$$

Then we separately calculate the CONS_local and CONS_entire:

(15) $$\mathrm{CONS}\_\mathrm{Entire}=\frac{N*\bar{d}*\omega }{2}$$

(16) $$\mathrm{CONS}\_\mathrm{Local}=\frac{N*\bar{d}*\omega }{2}*\frac{S}{S_{Area}}$$

Putting Equations (15) and (16) into Equation (14), we then obtain Theory 3. □

## 7. Experimental Evaluations

### 7.1. Evaluation on the Propagation Model

In this section, simulation results were presented in order to validate the performance of our proposed mathematical model and defending strategy for the mobile sensor worm. Detailed experimental parameters are shown in the Table 1. The basic attributes of the networks are listed in Table 2. All the experiments were conducted on a server running Microsoft Windows 7 with 2 CPUs and 16 GB of memory. The implementation was done in Visual Studio C++ 2012 and MATLAB 2012. The random numbers were produced by the C++ TR1 library extensions. The simulation results were averaged over 100 runs. The number 100 came from the discussion “How many runs are necessary before obtaining an average result” in [38].

For comparison, we developed a simulator DT-S (discrete-time simulator) [38] and considered its results as the real infection results. The DT-S, a classic propagation simulator, is widely adopted in experiments on worm/virus propagation. Its basic principle is that at any discrete time point, all nodes check their own security states. If the node is infectious, it spreads the worm to all of its neighbors with a certain probability and then loses the infectivity. Another classical worm propagation model proposed by Tang et al. [26] is considered as the contrastive model, which is based on epidemic theory. 

**Figure 6:**
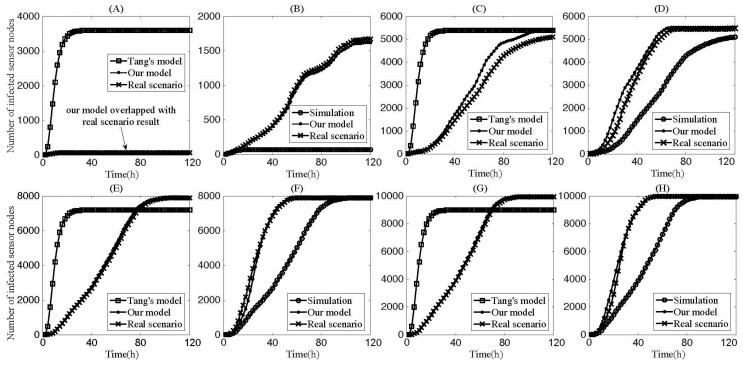
Number of infected nodes vs. propagation time when no defense strategies are implemented. The network sizes of (**A**,**B**) are 4000; of (**C**,**D**) are 6000; of (**E**,**F**) are 8000; and of (**G**,**H**) are 10000. Additionally, (**A**,**C**,**E**,**G**) are circumstances with only static sensor worm while (**B**,**D**,**F**,**G**) are circumstances with the mobile sensor worm.

Figure 6 shows that the number of infected sensors changed with the increase in infection time under different density settings. Generally, our model was consistent with the actual infection scenario generated by the DT-S. In Figure 6A, we observe that the real infection was not comparatively successful, and the infection number was about 100, accounting for 2.5% of the total sensors. Table 2 shows when the network size was 4000, due to low sensor density, there were 126 independent sensors that had no links. In reality, these independent sensors and a number of low-degree sensors made worm propagation difficult and prone to failure. Space restriction was a natural limitation for the worm infection process in WSNs. Epidemic models did not satisfy this property because they assumed that every sensor entity in the networks have an equal chance of coming into contact with every other entity per unit of time. As shown in Figure 6A, because it was not able to identify failure in the process of the worm propagation, Tang’s model misjudged the infection scale to an absurd extent. In Figure 6B, with the aid of the mobile worm carrier, the infection avoided the propagation limitation caused by a low deployed node density. In both cases, the results obtained by the proposed micro-mathematical model were rather consistent with the real infection. 

**Figure 7:**
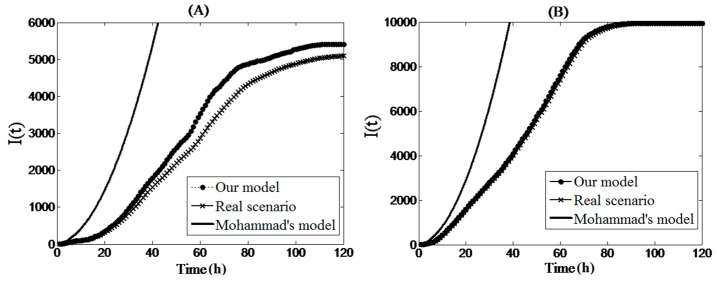
Our mathematical model vs. Mohammad’s individual boundaryless model (the network sizes of (**A**), (**B**) are 6000 and 10,000); I(t) means the number of infectedsensor nodes.

With the increase in density of the sensors, the infection scale rose, accordingly, from the results in Figure 6C to the results in Figure 6H. Meanwhile, the proposed mathematical model maintained better and more accurate experimental results for estimating the worm infection scale. Although the infection scenarios were distinct from each other, the common denominator between them was that the mobile worm carrier significantly improved the infection ability of the worm. Through these four groups of experiments, Tang’s model, based on the epidemic model, did not apply well to WSANs which were limited by the geographical space, in particular when sensor node density was quite low. Although node density was sufficiently high in Figure 6E,G, the infection peak values of Tang’s model were both lower than the peak values of the real infection. On the contrary, the proposed microscopic model was successful in achieving results close to the real worm infection results and thus could be applied to large WSANs. Figure 7 shows the numerical comparison of our proposed microscopic model and Mohammad’s individual boundaryless model. Similarly, our method is better than Mohammad’s model. In reality, Mohammad et al. optimized their method and proposed a bounded model. However, the bounded model is closely related to the shape of the WSNs and also cannot deal with the propagation scenario with a mobile worm carrier. 

**Figure 8:**
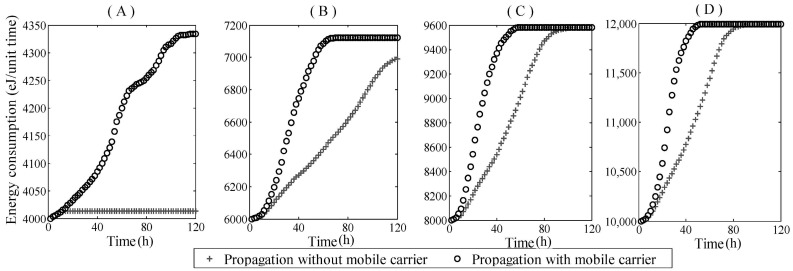
Network energy consumption vs. Propagation time (The network sizes of (**A**–**D**) are 4000, 6000, 8000, and 10,000, respectively).

Figure 8 shows the simulation results for network energy consumption per unit time after the sensor worm (static or mobile) began to spread. The network sizes of Figure 8A–D were 4000, 6000, 8000, and 10,000, respectively. Once the sensor worm propagated, the network energy consumption exponentially increased until all sensors in the network were infected. Therefore, it was necessary to interfere, and took control measures in the early stages of propagation to minimize cost.

In addition, a comparison of mobile and static sensor worm propagation shows that the worm carrier’s mobility has significantly accelerated the consumption of network energy. Specifically, from Figure 8B–D, when the propagation time reached sixty and the infection number caused by the mobile sensor worm arrived at the peak value, the energy consumption in the network with the mobile sensor worm was about twice as much as that in the network with the static sensor worm. The mobile sensor worm carrier had largely increased the infection number at the corresponding moments. Moreover, in Figure 8A, the effect of static worm propagation on energy consumption was very small; worm propagation had failed due to low node density. However, the mobile sensor worm overcame this limitation and increased energy consumption. In sum, the mobile worm carrier can greatly accelerate energy consumption and consumption velocity, which means that it is more dangerous than the static sensor worm.

### 7.2. Evaluation on the Defending Strategy

Our defending strategy, LDS, is a mixed method that includes both the blocking and patching processes. Compared to previous single immunization or patching operations, the proposed LDS may be more effective for defending against the worm. In reality, our method has better expansibility because it can be applied to distinct worm propagation scenarios as long as we can accurately detect the worm source. In this section, we evaluate our strategy based on simulation experiments.

There are a number of classical immunization algorithms, including random immunization, acquaintance immunization and target immunization. The random immunization method randomly selects a certain proportion of the nodes and performs an immune operation on them. Another immunization mechanism is to immunize a certain proportion of nodes with a greater degree in the network, which is generally referred to as the acquaintance immunization method. A type of tradeoff between the above two algorithms, the target immunization method, first, randomly chooses a certain proportion of nodes and then performs an immunization operation on the nodes with the largest degree in the neighbors of the selected nodes. For convenience, the random immunization method is referred to as RI, the acquaintance immunization method is referred to as AI, and the target immunization method is referred to as TI. These algorithms, all taking the degree characteristic as a measurement standard, and widely adopted in both industry and academia, are considered as contrast algorithms in our experiments. 

**Figure 9:**
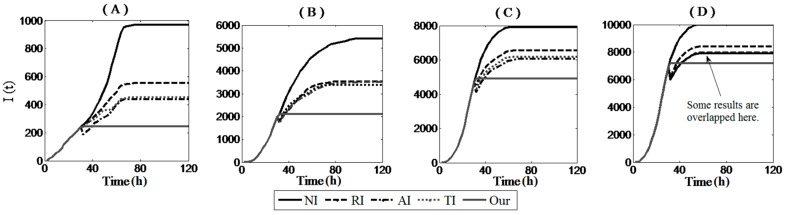
Number of infected nodes vs. propagation time when there are only immunization strategies, but no patches are implemented. The network sizes of (**A**–**D**) are 4000, 6000, 8000, and 10,000, respectively. We assume immunity rates are 20% and the immunization methods are executed at time 30.

Figure 9 shows the effect of different immunization methods under a diverse setting of deployment densities. First, we found that with no defense strategies, infection proportions in the four networks with different node densities were 25%, 87%, 100%, and 100%. If the network owner detected the worm and carried out immunization operations before all sensor nodes were compromised, the infection process slowed down and stabilized on a smaller value. In Figure 9A,B, experimental results show that our immunization strategy outperformed existing classic immunization algorithms by approximately 50% on average. In Figure 9C,D, our strategy outperformed other algorithms by 16% and 11% on average, respectively. Note that, there were some declines in the infection number when implementing the RI, AI, and TI because a number of infected sensor nodes were considered immunized sensor nodes, which led to a reduction in the number of infected nodes. Based on the experiments, we proved that it was more effective to immunize key topology links than immunize the important nodes. 

**Figure 10:**
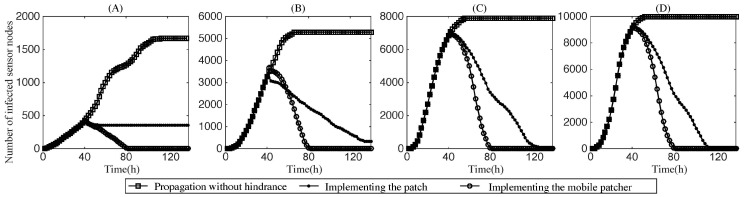
Number of infected nodes versus the propagation time when both the blocking and patching are implemented. In our experiments, we assume the defense strategies are executed at time 40. The network sizes of (**A**–**D**) are 4000, 6000, 8000, and 10,000, respectively.

Figure 10 shows the change in the infection number along with the simulation time when immunization and patching operations were both involved at time 40. In the experiments, we designed two employment patterns for the patch. In one, the patch was started from the initial infection location; in the other one, we utilized an actuator element to assist the patch packets’ diffusion. Figure 10A,B show that when the node density of the network was quite low and the mobile carrier was not considered, the diffusion of the patch failed, and the infection of the entire networks were difficult to recover. Ultimately, there remained infected sensor nodes that could not be repaired by the patch on time, as the number of running sensor nodes was too small and the network was not connected well. The mobile actuator could make up for this defect by moving to repair the infected sensor nodes. From Figure 10A–D, a mobile patcher repaired the infected networks faster and extensive simulation validated the robustness and efficiency of our worm defending scheme.

## 8. Conclusions

Worm attacks with mobile carriers may be a source of great danger in WSANs. However, traditional worm propagation models and defense strategies have rarely take them into consideration. In this paper, we modeled the spreading dynamics of a mobile sensor worm from the microscopic point of view. The model showed that the mobile carrier can appreciably accelerate worm dissemination. To this end, we proposed a local defending strategy LDS with a mobile patcher to recover the infected sensors within a restricted infection region and, thus, minimize the cost. Moreover, theoretical analysis and extensive experimental results demonstrated the effectiveness of both the proposed propagation model and defending method, which can be applied to energy-limited WSANs.

## Figures and Tables

**Table 1:** The parameters of experiments.

Parameter (Unit Symbol)	Value	Parameter (Unit Symbol)	Value
The number of sensors N	4000–10,000	Infection delay *α* (min)	1
The measure of area S (m^2^)	300 × 300	The locations of the actuator when it is infected (*x*_0_, *y*_0_)	(150, 150)
Communication radius *r* (m)	5	Infection rate *β*	0.9
Direction delay of actuator *τ* (min)	2	The time of mobile diffusion *t* (min)	0~120
Moving speed of actuator *v* (m/s)	1	——	——

**Table 2:** Basic attributes of networks.

	Attributes	Number of Links	Average Degree	Max Degree	Number of Independent Nodes
Network Size					
4000		14,030	3.51	13	126
6000		31,780	5.30	15	38
8000		56,232	7.03	19	9
10,000		88,360	8.836	22	4
